# Adaptive observed-based backstepping control for quantized robot arms

**DOI:** 10.1038/s41598-025-34449-7

**Published:** 2026-01-03

**Authors:** Zhuoxing Du, Hongyu Tang, Sufang Gao, Yuchen Cai

**Affiliations:** 1 School of Energy and Constructional Engineering, Shandong Huayu University of Technology, Dezhou, 253034 China; 2 School of Civil Engineering, Chongqing University, Chongqing, 400045 China; 3 BGI Engineering Consultants Ltd, Beijing, 100038 China

**Keywords:** Robotic arms, Signal quantization, State observer, Backstepping, Engineering, Mathematics and computing, Physics

## Abstract

Most existing control studies of robot arms are restricted to nonlinear systems but overlook real-time signal transmission. To resolve this issue, a novel observer-based backstepping control scheme, where both the input and output signals are quantized before transmission. Since quantized outputs cause discontinuities in the virtual controllers, auxiliary intermediate controllers are first designed using unquantized signals. By substituting quantized states into these controllers, the actual torque controller is obtained. Lemma 5 is introduced to handle quantization errors. And a novel quantized state observer with *n*-dimensional states is devised to evaluate the unmeasurable states. The validity of the presented scheme is verified via an example.

## Introduction

Robotic arms have garnered significant attention over the past few decades, and their control problems have been extensively studied in various practical engineering fields, such as industrial manufacturing^1^, rehabilitation^2,3^, and human-robot interaction^4^. To enhance the good control performance of the robot arms, advanced controller designs have been formulated to tackle issues including external disturbance, high nonlinear dynamics, and parameter uncertainties. In the actual engineering applications, robots are affected by a great many uncertainties (i.e.,the parametric, matched and unmatched model uncertainties). These problems make it difficult to acquire exact robot models. To this end, some well-known adaptive robot controllers, such as adaptive backstepping technique control^5–7^ and adaptive sliding mode control^8–10^ have been widely applied to address the motion with parametric uncertainties. Among them, an adaptive command-filter-based backstepping control approach for robotic manipulators was proposed by^11^, which is able to handle the parameter uncertainties and avoid the issue of “explosion of complexity”. An important point is that the above-mentioned references^5–11^ assume that nonlinearities satisfy growth conditions or are linearly parameterized. To remove these assumptions, some adaptive fuzzy logic system (FLS)/neural network (NN) schemes for unknown nonlinear robot dynamics have been proposed; see for examples^12–20^. It is noted that accurate information transmission for the robust systems’ controllers^12–20^ needs to be guaranteed. Nevertheless, due to the bandwidth constraints of communication channel, achieving this in networked control systems presents a significant challenge.

To tackle the problem of information transmission in remote control, quantization mechanism proposed by^21^ is an effective tool. The signal quantization, which can be commonly regarded as the mapping from continuous signals to discrete finite sets, has received much interest over the past few decades. It introduces obvious nonlinear characteristics that may result in instability or systems’ performance degradation. Thus, to ensure the system’s stability, it is crucial to yield sufficient precision and ensure relatively low communication rates. Recently, many significant outcomes about adaptive quantized control have been acquired^22–27^. Among them, for nonlinear systems with quantized input, a new adaptive compensation methodology is proposed in the literature^24^ to obtain desired tracking performance. On the basis of^24^, an event-triggered quantized control strategy was presented for flexible single-link arm systems in^25^. For multiple robot arms under input quantization, an observer-based adaptive prescribed-time controller was designed by^26^. Additionally^22–27^, only centred at robot systems under input quantization, yet their controllers remain reliant on continuous output/states. In actual robot systems, both the input and output/state signals are required to be quantized prior to communication.

To better satisfy the demands of real-world engineering, by using the dynamics filtering technique, Choi *et* *al*. introduced a recursive quantized backstepping-based feedback control approach for nonlinear strict-feedback systems with time-varying delays^28^ and without time delays^29^. Inspired by^29^, the problem of the quantized control being solved for nonlinear multiagent systems under state and input quantization in^30^. Although the literature^28–30^ obtained remarkable results, the states of their system need to be measurable. To this end, for nonlinear systems under output quantization, the problem of immeasurable states has been studied in^31,32^. In addition, the inverse optimal fuzzy control strategy for systems with output quantization was represented by^33^. It is mentioned that the systems’ states of the above literatures^31–33^ are 1-dimensional. However, in the robotic arms, each system’s state is composed of *n* components, which is more complicated than the above systems. As far as we are aware, there have been no reported studies on adaptive fuzzy control for robot systems with output and input quantization.

In light of the previous discussion, a fuzzy adaptive control for multi-link robotic arms under output and input quantization is investigated. Notably, quantized output and input signals of robotic arms make it challenging to handle this control problem. There are two primary difficulties: Firstly, different from the existing control studies of robotic systems, the signal quantization in both the output and input channels is considered in this research. So the systems receive output information in a discrete form, making the traditional backstepping technique directly inapplicable. How can the control process be designed to derive the actual control law? Secondly, the current state observers under output and input quantization^31–33^ have only been applied to systems with 1-dimensional states, so their observers are unsuitable for systems with *n*-dimensional states. How can an observer be designed to estimate the unmeasurable states?

The core contributions are shown below:

1. Due to the quantized output’s discontinuity, the partial derivatives of the virtual controllers are non-existent. To address this problem, initially, virtual intermediate controllers are devised. Subsequently, by replacing the continuous output with quantized output, both the actual torque controller and the intermediate controller are derived. Lastly, to cope with quantization errors, Lemma 5 is established.
2. Compared with the available studies for robot manipulators with input quantization^25,26^, both the output information and the input signals are quantized before communication. To estimate the *n*-dimensional immeasurable states, a novel high-dimensional quantized state observer is established.

The structure of this article is as follows: Section “Relevant knowledge and problem description” presents the necessary background knowledge and formulates the problem. A novel adaptive fuzzy output feedback controller design for robot systems is introduced in Section “Adaptive fuzzy output feedback controller design”. Stability analysis is provided in Section “stability analysis”. A simulation example is given in Section “A simulation example”, and the article concludes with Section “Conclusion”.

## Relevant knowledge and problem description

### Relevant knowledge

#### Definition 1

^34^. The uniform quantizer is defined as

1 $$\begin{aligned} q(s)=\left\{ \begin{array}{ll} {\pounds }_isgn(s),\ \pounds _i-\frac{d}{2}<|s|\le \pounds _i+\frac{d}{2}\\ 0,|s|\le \pounds _0 \end{array}\right. \end{aligned}$$

where $\pounds _0=\frac{d}{2}$ dictates deadzone’s size for *q*(*s*). The parameters $\pounds _1=\xi _0+\frac{d}{2}$ and $\pounds _{i+1}=\pounds _i+d\ (i=1,\dots ,n)$ are defined with $d>0$ representing the quantization interval length. Moreover, the characteristic $|q(s)-s|\le \varrho _s$ is met with $\varrho _s=max\{\pounds _0,\frac{d}{2}\}$.

#### Lemma 1

^35^. For a stable matrix $\Lambda _1 \in \Re ^{n\times n}$, the following condition holds:

2 $$\begin{aligned} ||e^{\Lambda _1 }|| \le \sigma _1e^{-\sigma _2t}, \end{aligned}$$

where $\sigma _1=\sqrt{\lambda _{max}(\Lambda _2)/\lambda _{min}(\Lambda _2)}$ and $\sigma _2=1/\lambda _{max}(\Lambda _2)$; $\Lambda _2$ denotes a symmetric positive definite matrix and satisfies $\Lambda _1^T\Lambda _2+\Lambda _2\Lambda _1=-2E$.

#### Lemma 2

^36^. For arbitrary vectors $\alpha =[\alpha _1,\dots ,\alpha _n]^T$ and $\beta =[\beta _1,\dots ,\beta _n]^T$, one has

3 $$\begin{aligned} \alpha ^T\beta \le \tfrac{1}{2}\alpha ^T\alpha +\tfrac{1}{2}\beta ^T\beta . \end{aligned}$$

#### Remark 1

Lemma 2 represents the formulation of Young’s inequality in the matrices case, which will be utilized in (43).

#### Lemma 3

^37^. Let $\lambda _{min}({O})$ and $\lambda _{max}({O})$ represent the minimum and maximum eigenvalues of matrix *O*. Given that $e \in \Re ^n$, and ${O}\in \Re ^{n\times n}$ is a real symmetric positive-definite matrix, then the following relationship is valid:

4 $$\begin{aligned} \lambda _{min}({O})||e||^2\le e^T{O}e \le \lambda _{max}({O})||e||^2. \end{aligned}$$

### Problem description

By employing the Lagrangian formulation, the dynamic model of an *n*-DOF robot system is described as:

5 $$\begin{aligned} M(q)\ddot{q}+C(q,\dot{q})\dot{q}+G(q)+F(\dot{q})=q(u), \end{aligned}$$

where $q=[q_{1},...,q_{n}]^T \in \Re ^n$ stands for the vector of joint angular, while $\dot{q}$ and $\ddot{q}$ denote the joint velocity and acceleration vectors, respectively. $q(u) \in \Re ^{n}$ is the quantized control input torque, $n\in N$ indicates the number of links. The inertia $M(q) \in \Re ^{n\times n}$ assumed to be known, is symmetric and positive definite. In addition, $G(q) \in \Re ^{n}$ and $C(q,\dot{q}) \in \Re ^{n\times n}$ indicate the gravitational torque and centripetal and coriolis force of the robot, respectively.

#### Property 1

^38^. $\dot{M}(q)-2C(q,\dot{q})$ is skew symmetric and satisfies $\varpi ^T\left( \dot{M}(q)-2C(q,\dot{q})\right) \varpi =0$, where $\varpi \in \Re ^n$ is a nonzero vector.

**Objective.** This paper aims to design an effective torque controller for the robotic arms under signal quantization, ensuring that all signals in this arm system remain semi-globally bounded.

### Fuzzy logic systems

If the function $\hslash (\varsigma )$ is continuous and unknown, it can be estimated using fuzzy logic systems as follows:

$Rule_\prime$: If $\varsigma _1$ is $F_{1}^{\prime }$, $\varsigma _2$ is $F_{2}^{\prime }$, $\ldots$ and $\varsigma _n$ is $F_{n}^{\prime }$,

    then $$y_H$$ is $$H^{h}$$, $$(\prime =1,2,\ldots ,m)$$,

where $y_H\in \Re$ and $\varsigma =[\varsigma _1,\varsigma _2,...,\varsigma _n]^{T}\in \Re ^n$ represent the output and input of the FLS, respectively. *m* denotes the total number of inference rules. $H^{\prime }$ and $F_{i}^{\prime }$ denote fuzzy sets. Here, the membership function for the fuzzy set $F_i^\prime$ is denoted as $\mu _{F_{i}^{\prime }}(\varsigma _i)$ for $i=1,2,\ldots ,n$.

In light of the center-average fuzzification, product inference engine, and Singleton fuzzifier, the value of $y_H$ can be derived as

6 $$\begin{aligned} & y_H(\varsigma )=\frac{\sum _{\prime =1}^{m}\vartheta _{\prime }\prod _{i=1}^{n}\mu _{F_{i}^{\prime }} (\varsigma _i)}{\sum _{\prime =1}^{m} \left( \prod _{i=1}^{n}\mu _{F_{i}^{\prime }}(\varsigma _i)\right) }. \end{aligned}$$

Let

$$\begin{aligned} & \psi _{\prime }(\varsigma )=\frac{\prod _{i=1}^{n}\mu _{F_{i}^{\prime }} (\varsigma _i)}{\sum _{\prime =1}^{m}\left( \prod _{i=1}^{n}\mu _{F_{i}^{\prime }} (\varsigma _i)\right) } \end{aligned}$$

and $\vartheta ^*=(\vartheta _{1},\vartheta _{2},\ldots ,\vartheta _{m})^{T},\ \varphi (\varsigma )=(\psi _{1}(\varsigma ),\psi _{2}(\varsigma ),\ldots , \psi _{m}(\varsigma ))^{T}$. Thus, the formulation of FLS can be expressed as

7 $$\begin{aligned} y_\hslash (\varsigma )=\vartheta ^{*T}\varphi (\varsigma ). \end{aligned}$$

#### Lemma 4

^39^. Considering a continuous function $\hslash (\varsigma )$ defined on a compact set $\Omega \subset \Re ^m$, for any $\varepsilon >0$, there exists a FLS (7) that satisfies the following equality:

8 $$\begin{aligned} |\hslash (\varsigma )-{\vartheta ^*}^T\varphi (\varsigma )|\le \varepsilon . \end{aligned}$$

## Adaptive fuzzy output feedback controller design

By defining $z_1=[z_{11}, \dots ,z_{1n}]^T=q$, $z_2=[z_{21},\dots ,z_{2n}]^T =\dot{q}$, $\bar{z}_2=[z_1,z_2]^T$, so the state-space model of (5) is expressed as a strict-feedback system

9 $$\begin{aligned} \left\{ \begin{array}{@{}l@{\;}l@{}} \dot{z}_1 =& z_2 \\ \dot{z}_2 =& M^{-1}(z_1)\big [q(u)-C(\bar{z}_2)z_2-G(z_1)-F(z_2)\big ] \\ y =& z_1 \end{array} \right. \end{aligned}$$

### State observer design

By considering the quantized input *q*(*u*) and output *q*(*y*), a state observer is designed to estimate these unmeasurable states. As shown below:

10 $$\begin{aligned} \left\{ \begin{array}{@{}l@{\;}l@{}} \dot{\hat{z}}_1 =& \hat{z}_2 + o_1\big (q(y) - \hat{y}\big ) \\ \dot{\hat{z}}_2 =& M^{-1}(q)\,q(u) + o_2\big (q(y) - \hat{y}\big ) \end{array} \right. \end{aligned}$$

where $\hat{z}_1$, $\hat{z}_2$, and $\hat{y}$ are the estimations of $z_1$, $z_2$ and *y*, respectively. Let $o_1, o_2 \in \Re ^{n\times n}$ denote the design parameters such that the subsequent matrix

$$\begin{aligned} A_c=\left[ \begin{array}{cc} -o_1 \ \ \ E \\ -o_2 \ \ \ 0 \end{array} \right] \end{aligned}$$

is a strict Hurwitz matrix, $E\in \Re ^{n\times n}$ denotes an identity matrix. Therefore, there exists a constant $d>0$ and a symmetric positive definite matrix *G* such that

11 $$\begin{aligned} A_c^TG+G^TA_c=-dE. \end{aligned}$$

Define an observation error $r=[r_1,r_2]^T$ with $r_i=z_i-\hat{z}_i,\ (i=1,2)$. Considering that unknown function terms $M^{-1}(z_1)[-C(\bar{z}_2)z_2-G(z_1)-F(z_2)]$, a FLS $\vartheta ^{*T}\varphi (\bar{z}_2)$ is utilized to approximate it and can be described as

12 $$\begin{aligned} \Phi (\bar{z}_2)=M^{-1}(z_1)\left[ -C(\bar{z}_2)z_2-G(z_1)-F(z_2)\right] =\vartheta ^{*T}\varphi (\bar{z}_2)+{\varepsilon }(\bar{z}_2). \end{aligned}$$

In the above expression, ${\varphi }=[{\varphi _{1}},\ldots , {\varphi _{n}} ]^T\in \Re ^{(n\cdot m)\times 1}$ is a basis function vector. $\vartheta ^{*T}=diag({\vartheta }_{1}^*,\vartheta _{2}^*,\dots \vartheta _{n}^*)\in \Re ^{n\times (n\cdot m)}$ is an optimal weight matrix with ${\vartheta _{s}}\in \Re ^{m} (s=1,2,\dots ,n)$ being a weight vector. Let ${{\varepsilon }}=({{\varepsilon }_{1}},\dots , {{\varepsilon }_{n}} )^T\in R^{n}$ be the approximation error vector, and ${{\varepsilon }}$ meeting ${\varepsilon }\le {\varepsilon }^*$, in which ${\varepsilon }^*$ denotes a constant.

In the light of (9), (10) and (12), let $o=[o_1,o_2]^T\in \Re ^{2n\times n}$, we have

13 $$\begin{aligned} \dot{r}=A_cr+o\left( y-q(y)\right) +\vartheta ^{*T}{\varphi }(\bar{z}_2)+{\varepsilon }. \end{aligned}$$

A Lyapunov function candidate can be written as $V_0=r^TGr.$ From (11), (13), $r_\prime =z_\prime -\hat{z}_\prime ,\ (\prime =1,2)$, Young’s inequality, and the characteristic of the quantizer $q(s)-s\le \varrho _s$. The derivative of $V_0$ holds

14 $$\begin{aligned} \dot{V}_0= & r^T(A_c^TG+G^TA_c)r +2r^TG\big [\vartheta ^{*T}\varphi (\bar{z}_2)+\varepsilon +o( y -q(y) )\big ]\nonumber \\\le & -d||r||^2 +3||r||^2+||G||^2\big (\vartheta ^{*T}\vartheta ^{*}\varphi (\bar{z}_2)^T\varphi (\bar{z}_2) +||\varepsilon ^*||^{2} +||\bar{o}||^2\varrho _y^2\big )\nonumber \\\le & -(d-3)||r||^2+\nabla _0, \end{aligned}$$

where $\nabla _0=||G||^2(\bar{\vartheta }^{*T}\bar{\vartheta }^{*T}+||\varepsilon ^*||^{2}+||o||^2\varrho _y^2)$, $\bar{\vartheta }^{*}=$sup$\{|{\vartheta }^{*}|\}$, and $\bar{o}=max\{||o_1||,||o_2||\}$.

#### Remark 2

The existing state observer designs for systems with output quantization in^31–33^ are only suitable for a system with 1-dimensional state. In this study, a high dimension state observer is designed, which contains the observer of robot systems with 1-dimensional states as a special case.

### Adaptive observed-based quantized torque control design

**Fig. 1:**
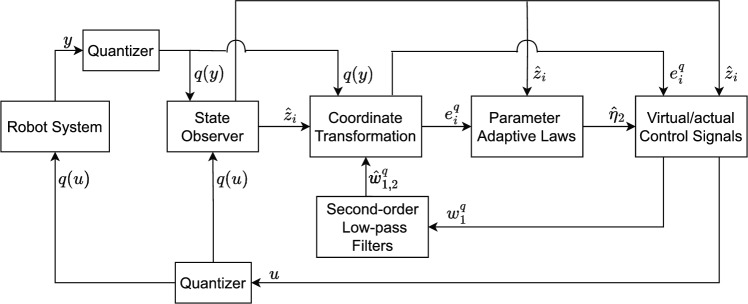
The flow chart of the quantized robot arms.

This part proposes a fuzzy adaptive control strategy for (9) under signal quantization. The control process will be divided into two parts, which will be specified later. This proposed strategy can effectively handle the discontinuity problem caused by state quantization and obtain the actual torque controller. Besides, Fig. 1 illustrates the block diagram of the robotic arms with output and input quantization.

(i) Making use of the original unquantized output signal *y* and the state estimate $\hat{z}_2$ obtained from the observer in (10), the following coordinate conversion is designed as:
  15 $$\begin{aligned} \left\{ \begin{array}{ll} e_1 =y \\ e_2 =\hat{z}_2-\omega _{1}^c \end{array}\right. \end{aligned}$$
  where $\omega _1 \in \Re ^n$ and $\omega _1^c\in \Re ^n$ are virtue control input and the filtered counterpart, respectively, and the filtering errors $\tilde{\omega }_{1,1}=\omega _1^c-\omega _1$. $e_1$ and $e_2$ represent the error variables. In addition, $\omega _{1}$ is a virtual stabilizing function, which will be designed in the ensuing part. $\omega _1^c$ is passed through the following second order command filter:
  16 $$\begin{aligned} \left\{ \begin{array}{ll} \dot{\omega }_1^c=\hat{\omega }_{1,2} \\ \dot{\hat{\omega }}_{1,2}=-2\zeta _1\aleph _1 \hat{\omega }_{1,2}+\aleph _1^T\aleph _1(\omega _1-\omega _1^c) \end{array}\right. \end{aligned}$$
  where $\omega _1^c(0)=\omega _1(0)$ and $\hat{\omega }_{1,2}(0)=0$, $\aleph _1 \in \Re ^{n\times n}$ is a natural frequency, and $\zeta _1 \in \Re ^{n\times n}$ is a damping ratio. Now let $\tilde{\omega }_1=[\tilde{\omega }_{1,1},\tilde{\omega }_{1,2}]^T\in \Re ^{2n\times 1}$, $\tilde{\omega }_{1,2}=\hat{\omega }_{1,2}\in \Re ^{n\times 1}$. According to (16), the derivative of $\omega _1$ is obtained as
  17 $$\begin{aligned} \dot{\tilde{\omega }}_1=A_1\tilde{\omega }_1+B\Xi _1, \end{aligned}$$
  where $B=[-E,0]^T\in \Re ^{2n\times n}$, and $\Xi _1=\dot{\omega }_1\in \Re ^{n\times 1}$. Because $\zeta _1$ and $\aleph _1$ are positive definite matrices,
  18 $$\begin{aligned} A_{1}=\left[ \begin{array}{ccc} 0 & E \\ -\aleph _{1}^{2} & -2\zeta _{1}\aleph _{1} \end{array} \right] \end{aligned}$$
  is a Hurwitz matrix. Hence, for any matrix $R_1>0$, there is a symmetric matrix $\bar{P}_1>0$ such that $A_1^T\bar{P}_1+\bar{P}_1 A_1=-R_1.$ **Step 1.** In the light of (9), (10), and (15), $\dot{e}_1$ is described as:
  19 $$\begin{aligned} \dot{e}_1=r_2+e_2+\omega _{1}+\tilde{\omega }_{1,1}. \end{aligned}$$
  Select the Lyapunov function candidate $V_1=\frac{1}{2}e_1^Te_1$. Moreover, by Young’s inequality, the following equation demonstrates the derivative of $V_1$:
  20 $$\begin{aligned} \dot{V}_1=e_1^T(r_2+e_2+\omega _{1}+\tilde{\omega }_{1,1}-\dot{q}_r)\le \tfrac{1}{2}e_1^Te_1+\tfrac{1}{2}r_2^Tr_2+e_1^Te_2+e_1\tilde{\omega }_{1,1}+e_1\omega _1. \end{aligned}$$
  Define an auxiliary intermediate controller $w_1$ as $\omega _1=-k_1e_1-e_1$ with $k_1>0$ denoting a constant. (20) can be further written as
  21 $$\begin{aligned} \dot{V}_1 \le -k_1e_1^Te_1 +\tfrac{1}{2}r^Tr+e_1^Te_2+e_1\tilde{\omega }_{1,1}-\tfrac{1}{2}e_1^Te_1. \end{aligned}$$
  **Step 2**. From the observer (10) and coordinate transformation (15), we have
  22 $$\begin{aligned} \dot{e}_2=M^{-1}(z_1)q(u)+o_2\left( q(y)-y\right) +o_2r_1-\hat{w}_{1,2}. \end{aligned}$$
  Select the Lyapunov function $V_2=\frac{1}{2}e_2^TM(q)e_2$. From (22), its derivative can be obtained:
  23 $$\begin{aligned} \dot{V}_2= & e_2^Tq(u)+e_2^TM(q)\left( o_2\left( q(y)-y\right) +o_2r_1-\hat{w}_{1,2}\right) \nonumber \\\le & e_2^T(q(u)-\tau )+e_2^TM(q)(o_2\varrho _y+o_2r_1-\hat{w}_{1,2})+e_2^T\tau . \end{aligned}$$
  Let $P_1(Z_1)=M(q)(o_2\varrho _y+o_2r_1-\hat{w}_{1,2})+e_1$ with $Z_1=[z_1,\hat{z}_1,\hat{w}_{1,2},\hat{w}_1^c]^T$. Similarly, a FLS $v^T\phi (Z_1)$ i.e., $P_1(Z_1)=\vartheta ^T\varphi (Z_1)+{\varepsilon }(Z_1)$ is applied to approximate it, where ${\varepsilon }(Z_1)\le {\varepsilon }^{*}$. In addition, a constant is defined as $\eta _2=||\vartheta ^T\vartheta ||$, $\hat{\eta }_2$ is the estimation of $\eta _2$, and $\tilde{\eta }_2=\eta _2-\hat{\eta }_2$ as the estimation error. Thus, the following equality is established:
  24 $$\begin{aligned} e_2^TP_1=e_2^T{\vartheta }^T{\varphi (Z_1)}\le \tfrac{1}{2a_2^2}{{{\eta }}}_2e_2^Te_2{\varphi (Z_1)}^T\varphi (Z_1)+\tfrac{a_2^2}{2}, \end{aligned}$$
  where $a_2>0$ is a designing constant. Design the following intermediate virtual controller $\tau$ as below:
  25 $$\begin{aligned} \tau =-k_2e_2-\tfrac{1}{2a_2^2}\hat{\eta }_2e_2\varphi (Z_1)^T\varphi (Z_1) \end{aligned}$$
  with a positive constant $k_2$. By incorporating (24) and (25) into (23), one obtains
  26 $$\begin{aligned} \dot{V}_2\le -k_2e_2^Te_2 +\tfrac{1}{2a_2^2}\tilde{\eta }_2e_2^Te_2\phi (Z_1)^T\phi (Z_1)+e_2^T\left( q(u)-\tau \right) -e_1^Te_2+\tfrac{a_2^2}{2}. \end{aligned}$$
(ii) To devise an actual actuator input torque and adaptive law for the arm system, we firstly define the surfaces and the error using quantized states as
  27 $$\begin{aligned} \left\{ \begin{array}{ll} e_1^q=z_1^q \\ e_{2}^q=\hat{z}_{2}-q(\omega _1^c) \\ \tilde{\omega }_{1,1}^q=q(\omega _1^c)-\omega _1^q \end{array}\right. \end{aligned}$$
  where $\omega _1^q=\omega _1^q(t),$ $\tilde{\omega }_{1,1}^q=\tilde{\omega }_{1,1}^q(t),\ q(\omega _1^c)=q(\omega _1^c)(t)$ are filtered errors and filtered signals, respectively. $\omega _1^q$ is a virtual control function and $e_1^q,e_2^q$ are error surfaces.

The second-order filters are utilized to compute the filtered signals as follows:

28 $$\begin{aligned} \left\{ \begin{array}{ll} q(\dot{\omega }_1^c)=\hat{\omega }_{1,2}^q \\ \dot{\hat{\omega }}_{1,2}^q=-2\zeta _1\aleph _1 \hat{\omega }_{1,2}^q+\aleph _1^T\aleph _1\left( \omega _1^q-q(\omega _1^c)\right) \end{array}\right. \end{aligned}$$

where $q(\omega _1^c)(0)=\omega _1^q(0)$, and actuator input torque $\hat{\omega }_{1,2}^q(0)=0$, $\aleph _1 \in \Re ^{n\times n}$ and $\zeta _1 \in \Re ^{n\times n}$ are positive constants.

Therefore, the intermediate controller $w_1^q$ and the actual control torque *u* are as follows

29 $$\begin{aligned} w_1^q= & -k_1e_1^q-e_1^q, \end{aligned}$$

30 $$\begin{aligned} u= & -k_2e_2^q-\tfrac{1}{2a_2^2}\hat{\eta }_2e_2^q\phi (Z_1)^T\phi (Z_1). \end{aligned}$$

For the convenience of writing, let $\rho _2=e_2^T\mu _2$ with $\mu _2=\frac{1}{2a_2^2}e_2\varphi (Z_1)^T\varphi (Z_1)$, the parameter adaptive law $\hat{\eta }_2$ on FLSs is designed as follows:

31 $$\begin{aligned} \dot{\hat{\eta }}_2=Proj[\Gamma _{\eta _2}\rho _2^q], \ \ \ \hat{\eta }_2(0)\ge 0, \end{aligned}$$

where $\rho _2^q=q(e_2)^T\mu _2, \mu _2^q=\tfrac{1}{2a_2^2}q(e_2)\varphi (Z_1)^T\varphi (Z_1),\ q(e_2)=e_2^q, \Gamma _{\eta _2}$ are positive parameters, for $\forall t\ge 0$, we have $||\hat{\eta }_2||\le \ell _\eta$.

#### Remark 3

By using the second-order command filtering-based backstepping method, we can effectively tackle the challenge arising from the repeated differentiability of virtual control laws, thus avoiding the “explosion of complexity” dilemma. Additionally, this method help design the actual controller.

## Stability analysis

**Fig. 2:**
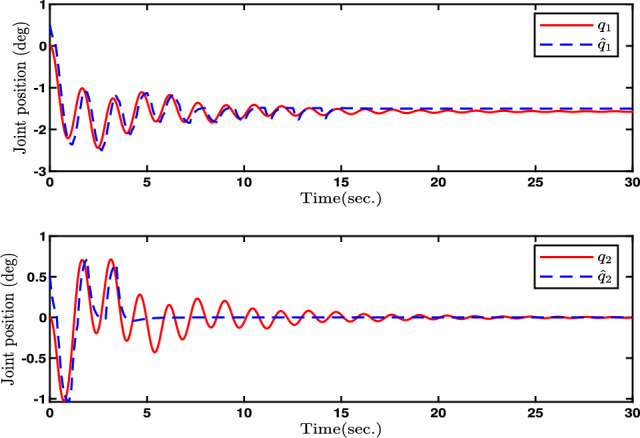
Evolutions of Joint position *q*(*t*) and its estimation.

In this part, Lemma 5 will be introduced to prove the boundedness of the errors between unquantized signals and quantized signals.

Before presenting Lemma 5, we first define the quantization errors:

32 $$\begin{aligned} & \Delta e_\imath =e_\imath -e_\imath ^q; \ \quad {} \ \ \Delta \mu _2=\mu _2-\mu _2^q; \ \quad {} \Delta \omega ^c_{1}=\omega ^c_{1}-q(\omega _{1}^c); \nonumber \\ & \Delta \omega _{1}=\omega _{1}-\omega _{1}^q; \ \ \ \Delta \hat{\omega }_{1,2}=\hat{\omega }_{1,2}-\hat{\omega }_{1,2}^q; \quad {} \Delta q=\tau -u, \end{aligned}$$

inside, $$\imath =1,2$$.

### Lemma 5

There exist positive constants $\gamma _{e_\imath }, \gamma _{\mu _2}, \gamma _{\omega _1}, \gamma _{\hat{\omega }_1},$ $\gamma$, and such that the above quantization errors (32) are bounded as follows:

33 $$\begin{aligned} |\Delta e_\imath |\le \gamma _{e_\imath };\ \ |\Delta \mu _{2}|\le \gamma _{\mu _2};\ \ |\Delta \omega _{1}|\le \gamma _{\omega _1};|\Delta \hat{\omega }_1|\le \gamma _{\hat{\omega }_1};\ \ |\Delta q|\le \gamma , \end{aligned}$$

where $$\hat{\omega }_1=[\omega _{1}^c,\hat{\omega }_{1,2}]^T$$

### Proof

(i) Since $\Delta e_1=z_1-z_1^q$, on the basis of the characteristics of norm and uniform quantizer, one obtains $||z_1-z_1^q||\le \varrho _y=\gamma _{e1}$. From the design of $\omega _1$ and $\omega _1^q$, we have
  34 $$\begin{aligned} ||\omega _1-\omega _1^q||=||-k_1(e_1-e_1^q)-(e_1-e_1^q)|| \le k_1\gamma _{e_1}+\gamma _{e_1} \triangleq \gamma _{\omega _1}. \end{aligned}$$
  From (32) and $\hat{\omega }_1=[\omega _{1}^c,\hat{\omega }_{1,2}]^T$, the definition of unquantized filters in (16), the quantized filters in (28), and $R_{\imath ,1}$ in (18), we can derive the following:
  35 $$\begin{aligned} \Delta \dot{\hat{\omega }}_1=A_1\Delta \hat{\omega }_1+\bar{B}\Delta \omega _1, \end{aligned}$$
  where $\bar{B}=[0,\aleph _1^T\aleph _1]^T$. By integrating both sides of (35), one has
  36 $$\begin{aligned} \Delta \hat{\omega }_1(t)=e^{A_1t}\Delta \hat{\omega }_1(0) +\int _0^te^{A_1(t-\iota )}\bar{B}\Delta \omega _1(\iota )d\iota . \end{aligned}$$
  Owing to the property of invertibility of $A_1$, the following inequality can be obtained:
  $$\begin{aligned} ||\Delta \hat{\omega }_1(t)||\le & \gamma _{\omega _1}||\bar{B}|| \ ||A_1^{-1}(E-e^{A_1t})|| + ||e^{A_1t}||\ ||\Delta \hat{\omega }_1(0)||. \end{aligned}$$
  On account of Lemma 1, we have $||e^{A_2t}|| \le \sigma _1e^{-\sigma _2 t}$, where $\sigma _1$ and $\sigma _2$ are positive constants. And ${\omega }_1^c(0)=\omega _1(0), \hat{\omega }_{1,2}(0)=0$, so $||\Delta \hat{\omega }_1(0)||=||\Delta \omega _1(0)||$. One obtains
  37 $$\begin{aligned} ||\Delta \hat{\omega }_1||\le \sigma _1||\Delta \omega _1(0)|| +\gamma _{\omega _1}||\bar{B}|| \ ||A_1^{-1}||(1+\sigma _1)\triangleq \gamma _{\hat{\omega }_1}. \end{aligned}$$
(ii) From the definition of $e_2$ and $e_2^q$, we have
  38 $$\begin{aligned} ||e_2-e_2^q||=||\hat{z}_2-{\omega }_1^c-\hat{z}_2+q({\omega }_{1}^c)||\le \gamma _{\hat{\omega }_1} \triangleq \gamma _{e_2}. \end{aligned}$$

Furthermore, one obtains

39 $$\begin{aligned} ||\mu _2-\mu _2^q||\le ||\tfrac{1}{2a_2^2}(e_2-e_2^q)|| \le \tfrac{1}{2a_2^2}\gamma _{e_2}\triangleq \gamma _{\mu _2}. \end{aligned}$$

According to (25) and (30), it yields

40 $$\begin{aligned} ||\tau -u||=||-k_2(e_2-e_2^q)-\hat{\eta }_2(\mu _2-\mu _2^q)|| \le ||k_2\gamma _{e_2}+\ell _{\eta }\Delta \mu _2||\triangleq \gamma . \end{aligned}$$

It is the complete proof of Lemma 5. $\square$

### Theorem 1

Consider the robot systems under output and input quantization. If the actual torque controller (30), the observer (9), the parameter adaptive law (31) and the virtual control law (29) are applied, all signals in this closed robot system are semi-globally bounded.

### Proof

Choose the subsequent Lyapunov function *V*,

41 $$\begin{aligned} V = V_0+V_1+V_2+\tfrac{1}{2\Gamma _{\eta _2}}\tilde{\eta }_2^2 +\tilde{\omega }_1^T\bar{P}_1\tilde{\omega }_1. \end{aligned}$$

In the light of (14), (17), (21) and (26), the time derivative of *V* can be computed as follows

42 $$\begin{aligned} \dot{V}\le & -\sum _{\imath =1}^2k_\imath e_\imath ^Te_\imath +e_1\tilde{\omega }_{1,1}-(d-\tfrac{7}{2})||r||^2 -\tfrac{1}{\Gamma _{\eta _2}}\tilde{\eta }_2\dot{\hat{\eta }}_2 -\tfrac{1}{2}e_1^Te_1+\left( -\tilde{\omega }_1^TR_1\tilde{\omega }_1 +2\tilde{\omega }_1^T\bar{P}_1B\Xi _1\right) +\nabla _0+e_2^T(q(u)-\tau )\nonumber \\ & +\tfrac{1}{2a_2^2}\tilde{\eta }_2e_2^Te_2\varphi (Z_1)^T\varphi (Z_1)+\tfrac{1}{2}a_2^2. \end{aligned}$$

Define the set $\prod$ as follows: $\prod =\{r^TGr+\tfrac{1}{\Gamma _{\eta _2}}\tilde{\eta }_2^T\tilde{\eta }_2+ {\sum _{i=1}^{2}e_i^Te_i} +\tilde{\omega }_1^T\bar{P}_1\tilde{\omega }_1\le \Upsilon \}$. Since $\prod$ is a compact set, $||\Xi _1||\le ||\bar{\Xi }_1||$ is designed on $\prod$, and $\bar{\Xi }_1$ is a constant.

On account of Lemma 2, we can deduce the following inequalities:

43 $$\begin{aligned} & 2\tilde{\omega }_1^T\bar{P}_1B\Xi _1 \le \tilde{\omega }_1^T\bar{P}_1BB^T\bar{P}_1^T\tilde{\omega }_1+\bar{\Xi }_1^2; \nonumber \\ & e_1^T\tilde{\omega }_{1,1}\le \tfrac{1}{2}e_1^Te_1+\tfrac{1}{2}\tilde{\omega }_{1}^T\tilde{\omega }_{1}. \end{aligned}$$

Since $R_1$ is positive definite, there exists an invertible matrix $\grave{R}_1$ such that $R_1=\grave{R}_1^T\grave{R}_1$. Thus, $\tilde{\omega }_1^TR_1\tilde{\omega }_1$ becomes

44 $$\begin{aligned} -\tilde{\omega }_1^TR_1\tilde{\omega }_1= & -\tilde{\omega }_1^T\grave{R}_1^T\grave{R}_1 \tilde{\omega }_1=-(\grave{R}_1\tilde{\omega }_1)^T\grave{R}_1\tilde{\omega }_1<0. \end{aligned}$$

By property 1, (43) and (44), one obtains

45 $$\begin{aligned} \dot{V}\le & -\sum _{i=1}^2k_ie_i^Te_i+\tfrac{1}{\Gamma _{\eta _2}}\tilde{\eta }_2\left( \Gamma _{\eta _2} \rho _2-\dot{\hat{\eta }}_2\right) +\tfrac{1}{2}\tilde{\omega }_1^T\tilde{\omega }_1 +\big (2\tilde{\omega }_1\bar{P}_1B\Xi -\tilde{\omega }_{1}^TR_1\tilde{\omega }_{1} \big )+e_2^T\left( q(u)-\tau \right) -(d-\tfrac{7}{2})||r||^2\nonumber \\ & +\tfrac{1}{2}a_2^2+\nabla _0\nonumber \\\le & -\sum _{i=1}^2k_ie_i^Te_i+\tfrac{1}{\Gamma _{\eta _2}}\tilde{\eta }_2\left( \Gamma _{\eta _2}\rho _2 -\dot{\hat{\eta }}_2\right) +\tfrac{1}{2}\tilde{\omega }_{1}^T\tilde{\omega }_{1} +\tilde{\omega }_1^T\bar{P}_1B B^T\bar{P}_1^T\tilde{\omega }_1+e_2^T\left( q(u)-\tau \right) +\nabla _1-(d-\tfrac{7}{2})||r||^2, \end{aligned}$$

where $$\nabla _1=\nabla _0+\frac{1}{2}a_2^2+\bar{\Xi }_1^2$$.

Consequently, from (31) and the property of the projection operator, we have the following formula:

46 $$\begin{aligned} \tfrac{1}{\Gamma _{\eta _2}}\tilde{\eta }_2\left( \Gamma _{\eta _2}\rho _2-\dot{\hat{\eta }}_2\right)= & \tfrac{1}{\Gamma _{\eta _2}}\tilde{\eta }_2\left( \Gamma _{\eta _2}\rho _2 -Proj\left( \Gamma _{\eta _2}\rho _2^q\right) \right) \le \tfrac{1}{\Gamma _{\eta _2}}\tilde{\eta }_2(\Gamma _{\eta _2}\rho _2-\Gamma _{\eta _2}\rho _2^q)\nonumber \\\le & \frac{\tilde{\eta }_2^2\gamma _{\rho _2}^2}{2\breve{d}_2^2}+\frac{\breve{d}_2^2}{2}, \end{aligned}$$

where $\breve{d}_2>0$ denotes a constant.

In addition, from the property of uniform quantizer and the definition of *u* and $\tau$, we can obtain

47 $$\begin{aligned} e_2^T(q(u)-\tau ) \le e_2^Te_2+\tfrac{1}{2}\varrho _u^2+\tfrac{1}{2}\gamma ^2. \end{aligned}$$

Considering Lemma 3, (46) and (47), we have

48 $$\begin{aligned} \dot{V}\le & -\tilde{k}_1e_1^Te_1-\tilde{k}_2e_2^Te_2-(d-\tfrac{7}{2})||r||^2+\nabla _2+\tfrac{1}{2\breve{d}_2^2}\gamma _{\rho _2}^2\tilde{\eta }_2^2 +\left( \tilde{\omega }_1^T\left( \tfrac{1}{2}E+\bar{P}_1BB^T\bar{P}_1^T\right) \tilde{\omega }_1\right) , \end{aligned}$$

where $$\tilde{k}_1=k_1,\tilde{k}_2=\frac{k_2}{\lambda _{min}(M(q))}, \nabla _2=\nabla _1+\frac{\check{d}^2_2}{2} +\frac{1}{2}\varrho _u^2+\frac{1}{2}\gamma ^2$$.

Consequently, one has

49 $$\begin{aligned} \dot{V} \le -CV+D, \end{aligned}$$

where $C=min\Big \{2\tilde{k}_1,2\tilde{k}_2, -\frac{\lambda _{max}(E+2\bar{P}_1BB^T\bar{P}_1^T)}{2\lambda _{max}(P_1)}, \frac{2d-7}{2\lambda _{max}(G)},$ $-\frac{2\check{d}_2^2}{2\Gamma _{\eta _2}\gamma _{\rho _2}}\Big \}$, $D=\nabla _2$. In (49), integrating both sides from $t_0$ to *t* ($t_0=0$) yields:

50 $$\begin{aligned} V(t)\le \frac{D}{C}+(V(0)-\frac{D}{C})e^{-Ct}. \end{aligned}$$

Therefore, Theorem 1 is proven. $\square$

### Remark 4

From (49) to (50), it can be observed that a larger gain *C* and smaller *D* improve control performance. Increasing ${k}_i$ and decreasing $\Gamma {\eta _2}$ result in a larger *K*. Additionally, raising $\Gamma _{\eta _2}$ leads to a reduction in *D*. However, excessively large values of ${k}_i$ may cause an increase in control amplitude, thereby raising energy consumption. Consequently, a balance must be struck between tracking accuracy and control energy efficiency.

## A simulation example

**Fig. 3:**
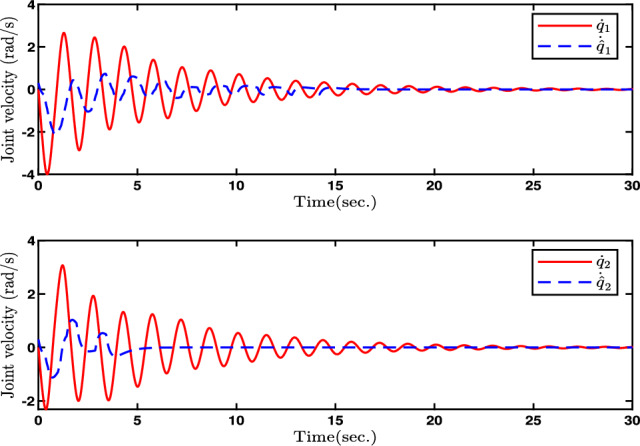
Evolutions of Joint velocity $\dot{q}$ and its estimation.

To assess the efficacy of the proposed approach, a two-DOF robot arm is considered as follows:

$$\begin{aligned} M(q)\ddot{q}+C(q,\dot{q})\dot{q}+G(q)=q(u), \end{aligned}$$

where the states’ vectors are described as $q=[ q_1, q_2 ]^T$, $\dot{q}=[ \dot{q}_1, \dot{q}_2 ]^T$, $F(q)=0$ for simplicity, and the dynamics of

$$G(q)=\left[ \begin{array}{ccc} G_1 \\ G_2 \\ \end{array} \right]$$, $$M(q)=\left[ \begin{array}{cc} m_{11} \ \ \ m_{12} \\ m_{21} \ \ \ m_{22}\\ \end{array} \right] ,$$

$$C(q,\dot{q})=\left[ \begin{array}{ccc} c_{11} \ \ \ 0 \\ 0 \ \ \ c_{22}\\ \end{array} \right] ,$$

are

$$\begin{aligned} \begin{aligned} \left\{ \begin{array}{ll} m_{11}=& \tau _{11}+2\tau _{22} \\ m_{12}=& \tau _{21}+\tau _{22} \\ m_{21}=& \tau _{21}+\tau _{22} \\ m_{22}=& \tau _{21} \\ c_{11}=& -\tau _{22}\dot{q}_2sin(q_2) \\ c_{22}=& -\tau _{22}(\dot{q}_1+\dot{q}_2)sin(q_2) \\ G_1=& 1/2cos(q_1+q_2)M_2gl_{g2}+\tau _{12}cos(q_1) \\ G_2=& 1/2M_2gl_{g2}cos(q_1+q_2) \end{array}\right. \end{aligned} \end{aligned}$$

where $g=9.8 m/s^2$ is the gravitational acceleration and $\tau _{i,j},\ i,j=1,2$ are design parameters given as

$$\begin{aligned} \begin{aligned} \left\{ \begin{array}{ll} \tau _{11}=& {1}/{2}M_1l_{g2}^2+M_2(l_{g1}^2+{1}/{2}l_{g2}^2) \\ \tau _{12}=& g({1}/{2}M_1l_{g1}+M_2l_{g1}) \\ \tau _{21}=& {1}/{2}M_2l_{g2}^2 \\ \tau _{22}=& {1}/{2}M_2l_{g1}l_{g2} \end{array}\right. \end{aligned} \end{aligned}$$

$M_1=2.5 kg$ and $M_2=10 kg$ denote the link mass, $l_{g1}=5\times 10^{-6}m,$ and $l_{g2}=1.5\times 10^{-4}m$ denote the mass center.

The experiment in this paper is conducted based on MATLAB R2020b and simulated in an NVIDIA 3060 GPU environment. For joint position *q* and joint velocity $\dot{q}$, over the interval $[-3,3]$, seven fuzzy sets are delineated with partitioning points at $f=-3,-2,\dots ,3$. Thus, the subsequent fuzzy membership functions are presented:

$$\begin{aligned} \hbar _\beta (\bar{Z})=exp[-\tfrac{1}{2}(\bar{Z}-f)^2], \end{aligned}$$

where $\beta =1,2,...,7$, so there are 7 rules in this FLS.

In the light of Theorem 1, the parameter adaptive law $\dot{\hat{\eta }}_2$, the virtual controller $w_1^q$, and the actual quantized controller *u* are implemented in the system (5) as follows:

$$\begin{aligned}&w_1^q&=-k_1e_1^q-\tfrac{1}{2}e_1^q;\\&u&=-k_2e_2^q-\tfrac{1}{2a_2^2}\hat{\eta }_2e_2^q\phi (Z_2)^T\phi (Z_2); \\&\dot{\hat{\eta }}_2&=Proj[\Gamma _{\eta _2}\rho _2^q]; \end{aligned}$$

and these control vectors have two components in this part. The system’s initial states are selected as $q(0)=[0,0]^T,\ \hat{q}(0)=[0.5,0.5]^T,\ \dot{q}(0)=[0,0]^T,\ \dot{\hat{q}}(0)=[0.3,0.3]^T$ and $\hat{\eta }_2(0)=0.5$. We select the design parameters $o_1=6,\ o_2=8, k_1=k_2=1,\ \Gamma _{\eta _2}=-1,\ a_2=1$. Besides, the parameters of the second order command filter can be elected as $\zeta _{1}=1/10E\in \Re ^{2\times 2},\ \aleph _{1}=15E \in \Re ^{2\times 2}$. $A_1$ is a Hurwitz matrix. By selecting $R_1=3E \in \Re ^{4\times 4}$ and solving $A_1^T\bar{P}_1+\bar{P}_1 A_1=-R_1$, a positive-definite symmetric matrix $\bar{P}_1$ can be derived as follows:

$$\begin{aligned} \bar{P}_1= \begin{bmatrix} \rho _{11} & \rho _{12} & \rho _{13} & \rho _{14} \\ \rho _{21} & \rho _{22} & \rho _{23} & \rho _{24} \\ \rho _{31} & \rho _{32} & \rho _{33} & \rho _{34} \\ \rho _{41} & \rho _{42} & \rho _{43} & \rho _{44} \end{bmatrix}, \end{aligned}$$

where $$\rho _{11}=\rho _{22}=113.02,\ \rho _{13}=\rho _{31}=\rho _{24}=\rho _{42}=0.0067,\ \rho _{33}=\rho _{44}=0.5022,\ \rho _{12}=\rho _{14}=\rho _{21}=\rho _{41}=\rho _{23}=\rho _{32}=\rho _{43}=\rho _{34}=0$$.

**Fig. 4:**
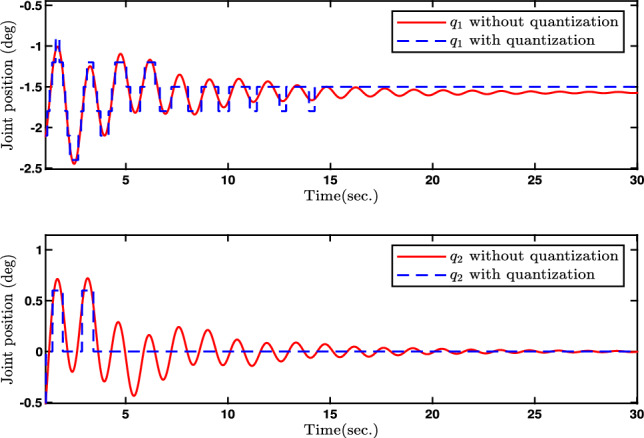
Evolutions of Joint position *q*(*t*) and its quantization.

**Fig. 5:**
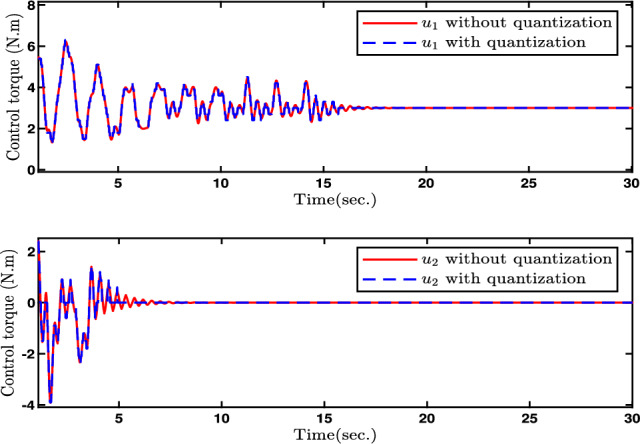
Evolutions of the control input *u*(*t*).

**Fig. 6:**
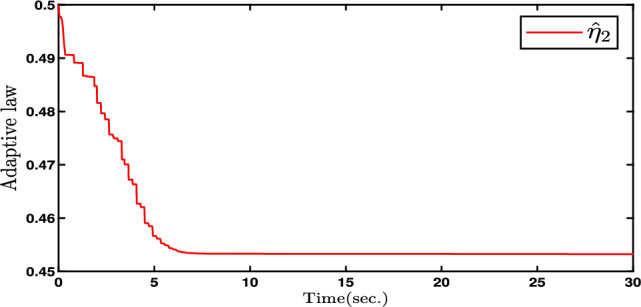
Evolutions of the parameter estimation $\hat{\eta }_{2}(t)$.

In this paper, Figs. 2, 3, 4, 5 and 6 denote the results of the simulation. Concretely, the trajectories of joint position *q* and its estimation are shown in Fig. 2. Figure 3 presents the joint velocity $\dot{q}$ and its estimation. Figure 4 illustrate the trajectory of joint position *q* with and without quantization. The evolution of the torque controller *u* and the adaptive parameter estimate $\hat{\eta }_2$ are represented in Figs. 5 and 6, respectively. In light of the above analysis, the desired control aim has been accomplished through the implementation of the proposed control method. This strategy can ensure the system (5) with n-dimensional states is infinite-time stable and every signal in the two-joint robot is semi-globally bounded.

## Conclusion

In this paper, every signal of input torque and output is quantized before communication. To avoid the partial derivatives of virtual controllers, the design of these controllers incorporates the command filtering technology and the backstepping technique. Then, by using substitution and the projection operator, a parameter adaptive law and a torque controller have been devised. Moreover, Lemma 4 has been introduced to compensate for the effects of quantization. Analyzing the outcomes of the simulation, the feasibility of this control strategy has been proved. The proposed strategy overlooks the effect of time delay, a common issue in physical devices, which can result in system instability, as highlighted in^40,41^. Therefore, our future research will focus on the control problem of time-delayed robot systems with input and output quantization.

## Data Availability

The datasets generated and/or analysed during the current study are available from the corresponding author on reasonable request.
